# BOOK NOTICES

**DOI:** 10.3402/ijch.v73.25561

**Published:** 2014-08-14

**Authors:** Frank Trovato, Anatole Romaniuk

Aboriginal Populations: Social, Demographic, and Epidemiological Perspectives. Frank Trovato & Anatole Romaniuk, editors. Edmonton, Canada: University of Alberta Press, ISBN-13: 978-0-88864-625-5 
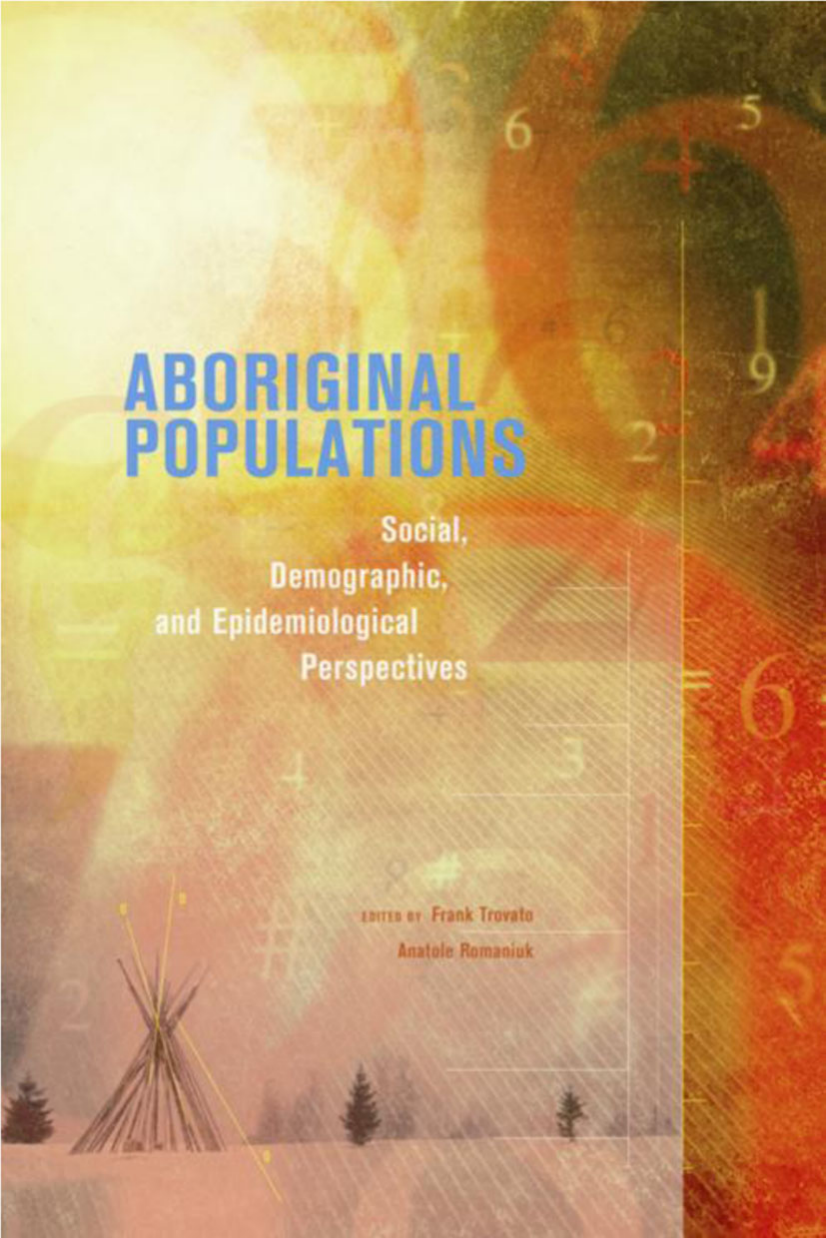


Experts from around the world review and extend the research on Aboriginal peoples in Canada, Australia, New Zealand, and the circumpolar North, mapping recent changes in their demography, health, and sociology and comparing their conditions with that of Aboriginal peoples in other countries.

Contributors point to policies and research needed to meet the challenges Aboriginal peoples are likely to face in the twenty-first century. This substantial volume will prove indispensable and timely to researchers, policy analysts, students, and teachers of social demography and Native Studies.

Order from: orders@gtwcanada.com

